# Possible Interrelationship of Inflammatory Cells in Dry Type Cutaneous Leishmaniasis

**Published:** 2017-04-01

**Authors:** Elham Taheri, Shahriar Dabiri, Manzumeh Shamsi Meymandi, Ebrahim Saedi

**Affiliations:** 1 *Dept. of Pathology, Pathology and Stem cell Research Center, Afzalipour Medical School, Kerman *

**Keywords:** Cutaneous Leishmaniasis, Macrophages, Dendritic Cells

## Abstract

**Background & Objective:**

There is a complicated interaction between leishmaniasis and the host immune cells, and also between the host immune cells. These interactions have fundamental effects on the outcome of the disease.

The current study aimed at characterizing the number, distribution, co-localization, and interrelation of 4 types of inflammatory cells in different clinical forms of dry-type cutaneous leishmaniasis (CL).

**Methods:**

Thirty-nine cases of CL were studied. The cases were classified clinically as 14 cases of acute leishmaniasis with indurated papules, nodules, and plaques with central crust formation < 2 years, 7 cases of chronic type with non-healing lesions > 2 years, and 12 cases of lupoid leishmaniasis with characteristic papules around previous scars of CL > 2 years. Paraffin-embedded blocks were stained with hematoxylin and eosin (H&E) and also stained immunohistochemically for CD4, CD8, CD68, and CD1a.

**Results:**

In acute CL, there was a significant correlation between CD68+ macrophages and CD1a+ epidermal dendritic cells (DCs); the population of CD68+ macrophages and CD1a+ epidermal DCs increased in parallel.

In lupoid CL, there was a significant correlation between CD1a+ epidermal DCs, and CD1a+ dermal DCs and population of CD1a+ epidermal DCs; the number of CD1a+ dermal DCs increased in parallel.

**Conclusions:**

The result of the current study could be used as a baseline to design and study the new targeted therapy of synergistic effects of macrophages and DCs to phagocytizing leishmania bodies; and/or suggestion planning of individualizing setup of vaccine by autologous interaction of macrophages and DC in CL.

## Introduction

Leishmaniasis is one of the most important parasitic disorders and a zoonotic disease characterized by parasitised mononuclear phagocytic system. It is considered a neglected disease by the World Health Organization (WHO), and is particularly common in tropical and subtropical regions (1). Cutaneous leishmaniasis (CL (is the most common form of leishmaniasis characterized by chronic skin lesions and permanent scars in the infected area (2).Epidemiological investigations indicated that 90% of all reports of CL occurred in Afghanistan, Algeria, Saudi Arabia, Iran, Sudan, and Syria (3,4).

At present, the diagnosis of CL is often made on the basis of epidemiologic and lesion characteristics, with the demonstration of the presence of the parasite in lesions (smear and Giemsa staining procedure), histopathological examination, isolation of Leishmania parasite from biopsies or aspirates in Novy-MacNeal-Nicolle (NNN) medium, serology, and molecular approaches (5).

It was previously proven that in CL the histopathological diagnosis varies from an inflammatory infiltrate of mononuclear cells and neutrophils, to granulomatous reactions with or without necrosis (6). 

Several investigations attempted to establish the importance of histopathology in the diagnosis of CL; however, different morphological findings were found. In this context, it is proved that the cellular immune response plays an important role in pathogenesis of CL (7-9). T lymphocytes are the main agents in lymphocytic infiltration and control of parasite replication in CL (10-12). It seems that parasite growth control is mediated by inflammatory process. The interaction of parasite-host and that of different inflammatory cells, and the correlation between inflammatory cells may affect parasite load in lesion and histopathological changes of lesion (13-15). Immunohistochemical (IHC) studies showed that population of inflammatory cells in CL included CD4+ and CD8+ T-Cells and B-Cells, macrophages, and natural killer cells (NKC) (16-17). Morgado et al., showed an association between the clinical course and the population of inflammatory cells in CL (18). 

The current study aimed at exploring the intercorrelation between different inflammatory cell types (CD4+ helper T-Cells, CD8+ cytotoxic T-Cells, CD68+ macrophages, and CD1a+ dendritic cells (DCs) in different forms of CL and describing the correlation between different inflammatory cells and histopathology of lesions.

## Material and Methods

The current study was approved by Ethics Committee of Kerman University of Medical Sciences. It was a retrospective case series study on the patients with CL diagnosed in Afzalipour Institute of Medical Sciences, Kerman, Iran. The patients were diagnosed using the established clinical, epidemiological, and histopathological criteria (19). Patients were identified by the natural history of their disease and the presence of single or multiple lesions. Clinical diagnosis was parasitically confirmed by Giemsa, or haematoxylin and eosin (H&E) staining of smears and biopsies, and/or polymerase chain reaction (PCR) assay. The patients were not under treatment at the time of study. Patients were divided into 3 groups based on the duration of disease: 

1) Acute leishmaniasis (<24 months: 14 patients)

2) Chronic leishmaniasis (> 24 months: 7 patients)

3) Lupoid leishmaniasis (presence of new lesions in the center or around the atrophic scar due to previous healed leishmaniasis: 12 patients)(20 -23).

Skin biopsies were embedded in 10% formalin and paraffin blocks were prepared. Sections with 4 micrometer thickness were obtained and H&E staining was done.

**Antibodies**

On all biopsies, in addition to H&E staining, 4 immunohistochemical markers were studied.The markers were monoclonal antibodies used for immunophenotypic characterization of leukocytes, directed to the following human cell surface antigens and included CD4 (code MU421-UC; clone 4B12), CD8 (code MU422-UC; clone1A5), CD1a (code MU490-UC; clone O10), and CD68 (code MU416-UC; clone KP1) (BioGenex company) ready to be used without dilution factor.

**Immunohistochemistry procedure**

Sections with 4 micrometer thickness were prepared. Slides were silanized to improve the adherence of tissue. After dewaxing and rehydrating paraffin sections, unmasking antigens with antigen retrieval was performed in 0.01 M citrate buffer in microwave oven at 800 W for 10 minutes. Slides were cooled down to room temperature. Slides were washed quickly in Tris-buffered saline PH 7.4. Slides stained with anti-CD4, anti-CD8, anti-CD68, and anti-CD1a antibodies and 3,3'-diaminobenzidine (DAB) chromogen. EnVision solution contain secondary antibody bind to the biotin and streptavidin bind to peroxidase were used.

**Leukocyte quantification**

Cells were counted using a light microscope (Olympus CX31) connected to a camera. All the fields of interest were counted in each section at a magnification of 400X, giving 2 to 4 × 10^4^ cells per section (24). Numbers of different inflammatory cells (cells/mm^2^) were determined by an ocular micrometer and manual hematology cell counter. Langerhans cells (LCs) (CD1a), helper T-Cells (CD4), cytotoxic T-Cells (CD8), and macrophages (CD68) were counted. Totally, 1950 fields of IHC stained sections (10 fields per slide for each CD4, CD8, and CD68 markers and 20 fields per slide for CD1a marker) were counted by micrometer.

**Azadeh classification**

Histopathological patterns based on Azadeh classification (23) included 4 groups: Class 1, anergic histiocytes with many leishman bodies; class 2, necrotizing granuloma with many plasma cells and few leishman bodies; class 3, organized epithelioid granuloma with few plasma cells and very occasional necrosis or leishman bodies; and class 4, necrotizing granuloma with stellate microabscess.

**Histopathological analysis**

The parameters of hyperkeratosis, parakeratosis, ulceration, acanthosis, exocytosis, abscess formation, spongiosis, apoptotic body, atrophy epidermis, melanophages collection, and pseudoepitheliomatous hyperplasia and congestion were reviewed based on previous studies of the histopathology of CL (25).

**Quantitative analysis**

Leishman body (parasite load) was reported in H&E sections with micrometer based on Ridley scoring system as follows: absent (0), scattered visible only in oil immersion (+), some visible in highly magnified slides (400×) (2+), many visible in mildly magnified slides (3+) (26). 

**Statistical analysis**

All the information was expressed as mean ± standard error of the mean (SEM). The means were calculated based on individual values for each patient. Comparisons between the groups were made by the non-parametric Mann–Whitney test and the Student *t* test for unpaired samples. The P-values < 0.05 were considered significant. The Kruskal–Wallis non-parametric test was used to analyze the variance to compare variability within groups. Correlations between variables were analyzed using Spearman rank correlation coefficient. All tests were performed using SPSS version 17 (SPSS Inc., Chicago, IL, USA).

**Results**

Thirty-nine patients were included in the current study; the mean age of the participants was 34.5 years (ranging from 15 to 55 years); 52.4% of the participants were male and 47.9% female. Acute CL was observed in 17(43.6%) cases, chronic CL in 8 (20.5%), and lupoid CL in 14 (35.9%); based on Azadeh classifications, 39.4%, 24.2% , 27.3%, and 9.1% of the cases were in class 1, class 2, class 3 and class 4, respectively. In quantitative analysis of leishman body on H&E sections, 30.3%, 15.2%, 12.1%, and 12.1% were found in classes 1, 2, 3, and 4 of Azadeh classifications, respectively.

**Immunohistochemistry results**

CD68+ macrophages were frequent (25.82±9.6 cells/mm^2^) and a significant correlation was observed between the number of them and lupoid form of CL (P <0.05).

The mean number of CD4+ helper T-Cells was 18.32±6.8 cells/mm^2^ and showed no correlation with the type of lesion. 

CD 8+ cytotoxic T-Cells with the mean number of 26.33±8.6 cells/mm^2^ had no significant relationship with the type of lesion.

CD1a+ LCs were observed in the epidermis (6.23±2.8 cells/mm^2^) with their projections forming a network. Positive staining for these cells was also detected in the dermis (2.8±1.5 cells/ mm^2^), but limited to the papillary dermis. These cells also had no significant relationship with the type of lesion.

The co-localization of these 4 types of cells in respect to type of lesion was analyzed.

**Fig 1:**
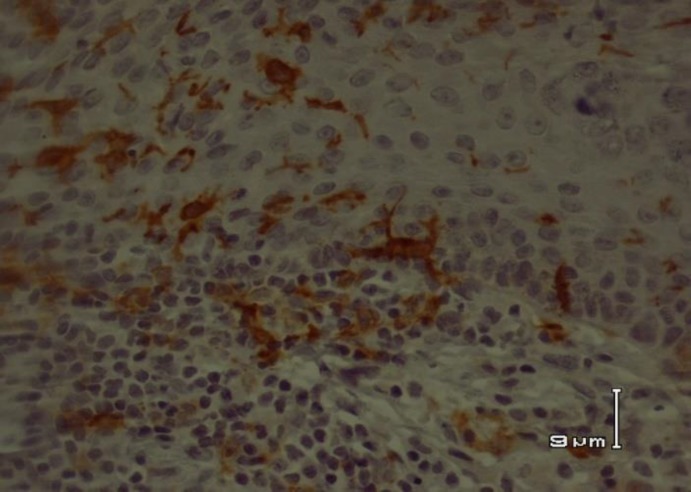
CD1a+ dendritic cells in acute cutaneous leishmaniasis

**Fig 2:**
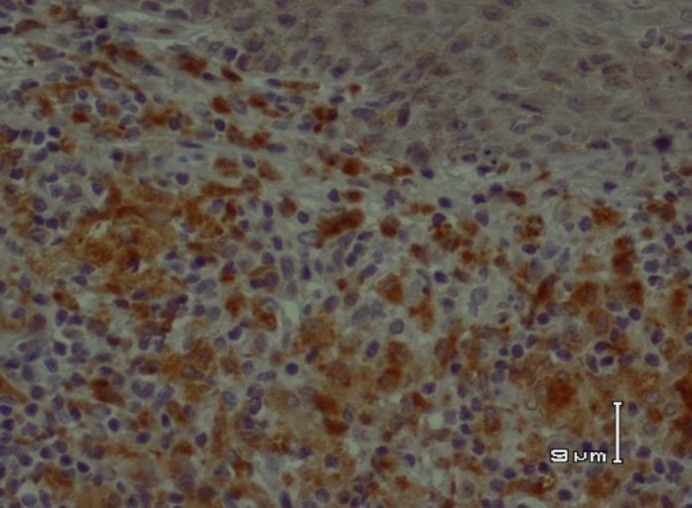
CD68+ macrophages in acute cutaneous leishmaniasis

 In acute CL, there was a significant correlation between CD68+ macrophages and CD1a+ epidermal DCs. Population of CD68+ macrophages and CD1a+ epidermal DCs increased in parallel (P =0.027) (Figures 1 and 2).

**Fig 3:**
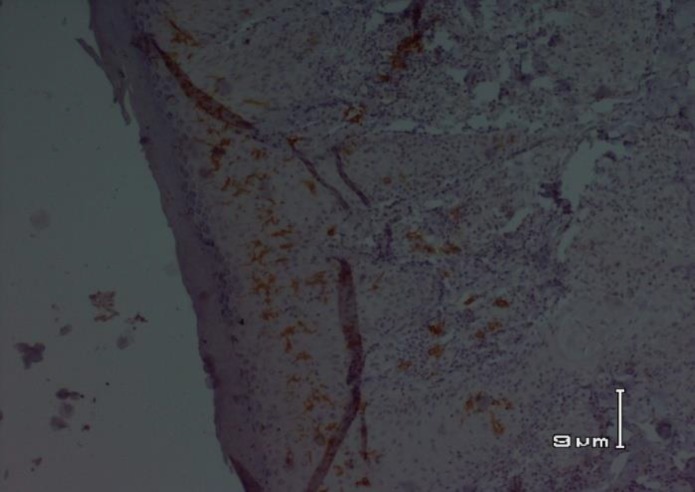
CD1a+ dendritic cells in lupoid cutaneous leishmaniasis

In lupoid CL, there was a significant correlation between CD1a+ epidermal DCs and CD1a+ dermal DCs. The population of CD1a+ epidermal DCs and CD1a+ dermal DCs increased in parallel (P =0.004) (Figure 3).

In all 3 forms, no other co-localization and interrelationship of inflammatory cells infiltrate were observed.

The possible correlation of CD4+ helper T-Cells, CD8+ cytotoxic T-Cells, CD68+ macrophages, and CD1a+ DCs with various Azadeh classifications was evaluated. The obtained results showed a significant association between the presence of CD68+ macrophages and class3 of Azadeh classification.

In quantitative analysis of leishman body on H&E slides, no significant association was observed between inflammatory cells and load of leishman body.

There was no significant association between hyperkeratosis, parakeratosis, ulceration, acanthosis, exocytosis, abscess formation, spongiosis, apoptotic body, atrophy epidermis, melanophages collection, and pseudoepitheliomatous hyperplasia and congestion with CD4+, CD8+, and CD68+ cells; while there was a significant correlation between spongiosis and dermal CD1a+ (P =0.013), and acanthosis and dermal CD1a+ (P =0.023). 

## Discussion

Leishmaniasis is caused by different species of genus Leishmania. Depending on the species, the parasite causes 3 different clinical presentations: cutaneous, mucocutaneous, and visceral leishmaniasis (27).

Cutaneous leishmaniasis (CL) is endemic in more than 98 countries, its overall prevalence is 12 million cases, and the annual incidence is 2 to 2.5 million cases (5). The parasite is transmitted by the bite of infected female sand flies, which inoculate the infective form of the parasite into the dermis of the host. Skin is the first point of contact with the protozoan, where the parasite is rapidly taken up by phagocytic cells. The skin is an important immune compartment that actively participates in host protection at both the early and later phases of infection. A wide variety of cells including neutrophils, macrophages, immature DCs, T lymphocytes and CLs provide considerable capacity to generate and maintain local immune reactions (8,28).

The current study evaluated the immunological and histological arrangements, and interrelationship of some important immune cells involved in the outcome of CL at the levels of acute, chronic, and lupoid forms .The inflammatory cells consisted of CD4+ helper T-Cells, CD 8+ cytotoxic T-Cells, CD68+ macrophages, and CD1a+ DCs.

As skin is an important site to transmit the leishmaniasis, the study on the immune responses of patients infected with CL and its association with distinct levels of leishman body load, and duration of CL allows new insights that elucidate the progressive or protective mechanisms of the infection (29).

The current study showed that acute CL had significant correlation between CD68+ macrophages and CD1a+ epidermal DCs and population of CD68+ macrophages and CD1a+ epidermal DCs increased in parallel during acute CL (P =0.027).

It is definite that Leishmania parasites interact and infect a variety of host cell types, CD68+ macrophages and CD1a DCs are the most important cells that regulate the outcome of infection. After the initial uptake of promastigotes by macrophages into the phagosome, subsequent fusion with lysosomes occurs, and the parasites should survive in this phagolysosomal environment. This is perhaps one of the most challenging environments for most pathogens, and Leishmania parasites are among the few protozoa that can survive and multiply in such an environment. Understanding how these organisms are able to survive and manipulate host cells to favor their replication and transmission is critically important to design new drugs or therapeutic strategies against the disease (30-33).

Leishmania spp. are obligatory intracellular pathogens; macrophages are mandatory for parasite survival. After initial infection, both neutrophils and macrophages are recruited to the lesion, and their interactions with the parasites significantly influence the outcome of infection (34,35). However, recent studies suggest that more neutrophils are recruited to the infection site and these cells are very efficient in early parasite uptake (36).

Macrophages that phagocytosed parasites are the last host cells for parasite replication and also the effector cells responsible for the destruction of parasites. Macrophages could be activated by different signals. Thus, suitable activation of macrophages is vital to eliminate leishmaniasis as an intracellular pathogen. The products of CD4+ T helper1 (Th1) cells and NK cells mediate classical activation of macrophages.

On the other hand, to evade killing by activated macrophages, Leishmania parasite should manipulate the macrophage activation pathways in a manner that favor their survival and proliferation. Interleukin (IL)-12 is a critical cytokine required for CD4^+^ Th1 development and production of IFN-γ (37). IL-12 is mainly produced by antigen-presenting cells (APCs), and macrophages were initially proposed as the major source of IL-12. There is evidence that macrophage ability to produce IL-12 is selectively impaired by the parasites (38-39).

DCs are hematopoietic bone marrow derived leukocytes that are widely distributed all over the body (40). DCs are specialized in uptaking, processing, and presenting antigens to T-Cells. Macrophages are also professional APCs and the main host to harbor Leishmania parasites and effector cells for parasite killing, but macrophages infected with Leishmania parasites do not secrete IL-12 (41) and, hence, are unable to stimulate antigen-specific CD4^+^ Th1 cell response (42). Indeed, several reports show a central role for DCs in organizing immune responses in leishmaniasis (41,43).

The current study showed that DCs and macrophages might regulate the outcome of leishmaniasis, which was consistent with the results of other studies.

Following the infection, both the macrophages and DCs phagocytose leishman bodies lead to different functional outcomes. Infected DCs produce IL-12, which is critical to develop IFN-γ producing CD4+ cells. IFN-γ affects the infected macrophage; leading to the activation (classical activation), and production of nitric oxide and other free radicals that are important for intracellular parasite killing (44,45). 

The current study showed that in lupoid CL, there was a significant correlation between CD1a+ epidermal DCs and CD 1a+ dermal DCs; and the population of CD 1a+ epidermal DCs and CD 1a+ dermal DCs increased in parallel (P =0.004).

DCs interdigitate the processes between epidermal keratinocytes, and comprise approximately 2% of the cell density of the epidermis. These cells are found in many epithelia, which are in contact with the external environment (46).

Meymandi et al. identified amastigotes of *Leishmania* spp. within the cytoplasm of intraepidermal DCs. In addition, transepithelial elimination of amastigotes of *Leishmania* spp. in CL was described (21, 47).

After the initiation of *L. major *infection, the local inflammatory response induces DCs to cross the dermoepidermal junction to allow phagocytosis of parasites in the dermis (20,44,48). 

The current study showed that predominant lymphocyte was CD8+ cytotoxic T-Cells supported by previous studies (25,40,50). 

CD8 T cells producing IFN-γ are important to modulate the CD4 T cell response.

The depletion of CD8 T-Cells did not interfere with the proliferative ability of CD4 T-Cells, but a reduction was observed in the percentage of CD4 T-Cells producing IFN-γ, and this effect was associated with an increase in parasite load in mice, suggesting an interaction between CD4 and CD8 T-Cells (51). In human leishmaniasis, important roles of CD8 T-Cells in the healing process through IFN-γ production (52) and in resistance to the infection are described (53).

The current study found a significant correlation between Azadeh class 3 lesions and CD68+ macrophages.

In the current study, the predominant cells in classes 1 and 2 of Azadeh classification were CD1a+ DCs, supported by other studies (21).

There was a significant relationship among dermal CD1a+ DCs, spongiosis, and acanthosis. As mentioned in the previous studies, cutaneous infection by *L. major* and resulting local inflammatory response induces DCs to cross the dermoepidermal junction to allow phagocytosis of parasites in the dermis (21,54), and this fact may result in the histopathological findings of the current study.

The current study characterized the inflammatory cells interrelation and co-localization of the infiltrate in different skin lesions of CL. In conjunction with the results of other investigations, the current study results provided strong evidence that LCs of epidermal origin may be one of the essential parts of an effective immune response against CL. Furthermore, the current study demonstrated that LCs traversing the dermoepidermal junction contain amastigotes of *Leishmania *spp. within the cytoplasm. The result of the current study can be used as a baseline to design and assess new targeted therapies and/or planning individualized set up of vaccine by self-interaction of macrophages and DC in CL.
